# Measuring School Climate among Japanese Students—Development of the Japan School Climate Inventory (JaSC)

**DOI:** 10.3390/ijerph17124426

**Published:** 2020-06-19

**Authors:** Tomoko Nishimura, Manabu Wakuta, Kenji J. Tsuchiya, Yuko Osuka, Hideo Tamai, Nori Takei, Taiichi Katayama

**Affiliations:** 1 Research Centre for Child Mental Development, Hamamatsu University School of Medicine, Hamamatsu, Shizuoka 431-3192, Japan; tsuchiya@hama-med.ac.jp (K.J.T.); tamaihideo@s04.itscom.net (H.T.); noritakei-psy@umin.ac.jp (N.T.); 2 Institute of Child Developmental Science Research, Hamamatsu, Shizuoka 430-0929, Japan; wakuta@ugscd.osaka-u.ac.jp (M.W.); osuka@kodomolove.org (Y.O.); katayama@ugscd.osaka-u.ac.jp (T.K.); 3 Department of Child Development and Molecular Brain Science, United Graduate School of Child Development, Osaka University, Suita, Osaka 565-0871, Japan; 4 Center for the Study of Child Development, Mukogawa Women’s University, Nishinomiya, Hyogo 663-8558, Japan; 5 Department of Psychosis Studies, Institute of Psychiatry, Psychology and Neuroscience, King’s College London, London WC2R 2LS, UK

**Keywords:** school climate, measurement invariance, quality of life, bullying, peer relationship problems

## Abstract

School climate is a significant determinant of students’ behavioral problems and academic achievement. In this study, we developed the Japan School Climate Inventory (JaSC) to see whether it measures school climate properly. To do so, we investigated whether or not the measurement with JaSC varies across sub-groups of varying grade and of gender and examined the relationship between the perception of school climate and the psychological and behavioral traits at individual levels in a sample of Japanese elementary and junior high school students (n = 1399; grade 4–9). The results showed that the measurement was consistent, since single-factor structures, factor loadings and thresholds of the items were found not to vary across sub-groups of the participants. The participants’ perception of school climate was associated positively with quality of life, especially in school (β = 0.152, *p* < 0.001) and associated negatively with involvement in *ijime* (bullying) as “victim” and “bully/victim” (β = −0.098, *p* = 0.001; β = −0.188, *p* = 0.001, respectively) and peer relationship problems (β = −0.107, *p* = 0.025). JaSC was found to measure school climate consistently among varying populations of Japanese students, with satisfactory validity.

## 1. Introduction

The earliest forms of the concept of school climate can be traced back more than 100 years. Perry [1] described how students are affected by the quality of their environment and highlighted the crucial influence of school culture or climate, on students’ outcomes. School climate typically refers to the quality and character of school life, with students, families and educators all contributing to the operation of a school and the care of the physical environment, making it a group phenomenon, larger than any one person’s experience [2]. In other words, students’ individual perceptions of school climate are aggregated into a school-wide climate, both of which affect students’ academic and behavioral outcomes [3].

Although there is no consensus on what elements make up the school climate, Cohen et al. [2] reviewed previous studies and outlined four major aspects—safety, teaching and learning, relationships and environmental-structural aspects.

Feeling safe in school is a fundamental need of all students [4]. School safety refers to an absence of crime and violence but also includes a supportive social environment, where students can feel safe from harassment, bullying and other acts of incivility or hostility [5]. A safe school climate has been shown to reduce bullying and violence. Eliot et al. [6] found that student perceptions of a supportive school climate are important for students to be willing to seek help from adults at school over bullying and threats of violence. Gottfredson et al. [7] reported that schools in which rules are effectively enforced or schools with better discipline management, have lower rates of student victimization and student delinquency.

An orderly learning environment and an adequate supply of material support for teaching have been found to be related to student achievement [8]. Fraser [9] reported that an adequate learning environment is consistently associated with students’ achievement and affective outcomes. A meta-analysis concluded that a positive school climate contributed to higher academic achievement and a decreased negative influence of poor socioeconomic status (SES) background characteristics and other risk factors on academic achievement [10].

The quality of social relationships within a school, has been found to be associated with good attendance and the increased mental and emotional wellbeing of students [11]. Bond et al. [12] reported that having both good school and social connectedness was associated with good mental health and academic outcomes in later years, while students with low school connectedness and good social connectedness were at elevated risk of anxiety/depressive symptoms.

Environmental and structural factors, such as cleanliness, adequate space and materials and the size of a school are important aspects of school climate [2]. Classroom layout, activity schedules and student-teacher interactions can also influence student behavior and feelings of safety [13]. McNeely et al. [14] found that the size of schools is negatively correlated with school connectedness, although reducing the school size is not the only way to improve the school environment [4].

A number of scales have been developed and validated for school climate research [3], many of which measure the students’ perception of school climate. In terms of invariability across populations, Bear et al. [15] confirmed that the scale they developed could be used across a wide range of grades (i.e., elementary, middle and high school), racial-ethnic groups and genders and demonstrated that the factor structure of the scale was stable across these sub-groups of students. The association of school climate as a group phenomenon, with each individual in the group contributing the school climate, has been an issue. Researchers have addressed this issue by investigating the relationship between students’ perception of school climate and individual psychological and behavioral traits, with the significant associations representing the external validity of the scales, as well as the relationship between school-wide climate and students’ academic and behavioral outcomes. For instance, Zullig et al. [16] revealed that higher levels of positive perceptions of school climate are associated with higher levels of positive perceptions of the quality of life, especially school satisfaction, at the individual level. Similarly, Hung et al. [17] reported a negative association between bullying victimization at school and perception of school climate. They also reported that emotional problems (e.g., anxiety and depressive mood) and conduct problems (e.g., lying and cheating) as measured by the Strengths and Difficulties Questionnaire (SDQ) [18] were negatively associated with an individual’s perception of school climate.

In Japan, research on school climate is limited. No attempt has been made to translate scales developed in other countries. This may be due to Japan’s own educational context, with its emphasis on school events and discipline [19]. In addition, in our previous attempts to translate scales developed in other countries, we found that several items did not have sufficient measurement precision because they involved content that rarely occur in Japanese schools. Thus, by extracting items from existing scales, we created a new school climate measure, the Japan School Climate Inventory (JaSC), covering assessment items available in the literature [20]. We took 32 items and confirmed that these items had satisfactory measurement precision (i.e., reliability). However, we have not yet determined whether the measure is valid in terms of consistency across sub-groups of sex (i.e., boys and girls) and grade level (elementary and junior high school students). Furthermore, we have not yet investigated the external validity of the scale—that is, the association between students’ perception of school climate and their psychological and behavioral traits that have been shown to be related to the perception of school climate.

In the current study, we aim to develop a scale (i.e., JaSC) that properly measures school climate among Japanese students. To do so, we (1) investigate whether the measurement of the scale is invariant across sub-groups of gender and grade levels and (2) examine whether the measured school climate, perceived at the individual level, is associated with students’ individual factors such as quality of life, bullying experiences in school and behavioral and emotional problems.

## 2. Materials and Methods

### 2.1. Participants

Four public schools (3 elementary and 1 junior high), located in a middle-sized industrial city in Japan, were recruited via the Board of Education. All the students (grades 4–9) enrolled in the regular classes of the school assigned to participate were included. Of total of 1462 students and their guardians invited, 1399 (95.7%) students and 1300 (88.9%) guardians responded. The characteristics of the participants are shown in Table 1.

The study was carried out in accordance with the Code of Ethics of the World Medical Association (Declaration of Helsinki) and approved by the Hamamatsu University School of Medicine and its University Hospital Ethics Committee (Ref. 14-384). The study’s purpose, significance and methodology were explained to students. They were also informed that they would not be penalized if they did not participate. Oral assent was obtained from the students and written consent was obtained from their parents or guardians.

### 2.2. Measurements

School climate—Students completed the JaSC, which consisted of 32 items. Briefly, the procedure to develop this scale was as follows. First, 101 items were taken from five existing scales of school climate—three scales that had been developed mainly in Western countries and two questionnaires used in Japan. The Western scales comprised the Inventory of School Climate-Student (ISC-S; 50 items) [21], the School Climate Measure (SCM; 48 items) [22] and the Delaware School Climate Survey-Student (DSCS-S; 23 items) [15]. The Japanese questionnaires, which have been used to assess the learning attitudes and school life of students, were the National Assessment of Academic Ability (National Institute for Educational Policy Research, Japan, 2016, 18 items) [23] and the Anti-Bullying Comprehensive Assessment Sheet (54 items) [24]. Based on suggestions by members of the expert advisory board (the “Kodomo-minna project” of the Ministry of Education, Culture, Sports, Science and Technology, Japan), 17 out of 101 items were excluded as they did not pertain to Japanese school life (e.g., “Has anyone offered or tried to sell you drugs at school?”, “Students help decide how class time is spent”). Then, with the item response theory model, the scale was reduced to 32 items on the basis of the measurement precision of the item (the item information function) and appropriateness of the response categories (category characteristic curves) [25,26]. We confirmed that the measurement of the scale was most precise in the range of −2 to 0 standard deviation from the mean along the trait profile (individual perception of school climate) and Cronbach’s alpha, as an index of reliability, at this range is approximately 0.97. In other words, a very high reliability was confirmed, especially for students who felt the school climate worse than the average respondents. In addition, items where a response category covered a very narrow or very wide range of the trait profile were dropped and we confirmed that response categories of each item included in JaSC appropriately represented the trait profile. There were five response categories for each item, from 0 = fully disagree to 4 = fully agree. The mean scores were calculated by totaling the responses to the individual items and dividing the sum by the total number of items (32 items), which we regarded as a perceived school climate for each of the participating students.

Quality of life—We used the Pediatric Quality of Life Inventory 4.0 generic scale (PedsQL) self-rating version to evaluate the physical and psychosocial quality of life of students [27,28]. The questionnaire was a five-point Likert scale and comprised 23 items. Scores on the physical, emotional, social and school functioning were obtained, with higher scores indicating that the child had a better quality of life.

Involvement in *ijime* (bullying)—We used the Japan Ijime Scale [29], which is a three-part self-report questionnaire consisting of a victimization subscale, a witnessing subscale and a perpetration item. Using students’ responses on victimization and perpetration, we categorized involvement in *ijime* as follows—victims (victimization only), bullies (perpetration only), bully/victims (both victimization and perpetration in the same individual) and not involved.

Strengths and Difficulties Questionnaire (SDQ)—Children’s emotional and behavioral problems were assessed using the parent-rated SDQ [18,30]. The SDQ has five subscales that cover children’s emotional and behavioral functioning (i.e., emotional symptoms, conduct problems, hyperactivity–inattention and peer relationship problems), as well as personal strengths (i.e., prosocial behaviors). Scores of each subscale were calculated.

Self-rated academic achievement—Students were asked about their own academic achievement, with a perfect score being 100 points.

Background characteristics—Students’ demographic data concerning grade and sex were obtained from students and data on household income was obtained from parents.

### 2.3. Statistical Analysis

#### 2.3.1. Consistency of Measurement across Sub-Groups (Measurement Invariance)

To investigate measurement invariance across sub-groups, we assessed the factor structure of the JaSC using exploratory factor analysis (EFA). We examined single- and multi-factor solutions where an eigenvalue exceeded 1.0. The geomin factor loadings and factor correlations of each solution were examined. After confirming that a single factor was the best solution, we probed measurement invariance within the framework of confirmatory factor analysis (CFA) to ascertain whether the factor structure and factor loadings could be reproduced in the different sub-groups (i.e., boys and girls; elementary and junior high school). A least restrictive invariance model (*configural* model) was initially specified, in which only factor structures were held constant across the two sub-groups and factor loadings, thresholds and residuals were estimated freely [31]. Next, the equality of the factor loadings between the two sub-groups was examined (*metric* model). After *metric* invariance was found, the *scalar* model was tested by constraining the factor loadings and thresholds to be equal across the sub-groups. Model comparisons were conducted using the χ^2^ test procedure. The *p*-value more than alpha level (*p* > 0.05) indicates for example, that a *metric* invariance model is not a significantly worse fit than the *configural* invariance model. However, the *p*-value in the χ^2^-test is likely to be influenced by the sample size. Stringent criteria have been recommended for evaluating models with large sample sizes and for testing loading and threshold invariances, a change of ≥ −010 in the comparative fit index (CFI), supplemented by a change of ≥ 015 in the root mean square error of approximation (RMSEA) indicates non-invariance [32].

#### 2.3.2. Association of the Measured School Climate with Students’ Individual Factors

To investigate the external validity of JaSC, we estimated the association of JaSC scores with other individual properties, including students’ quality of life, their involvement in *ijime*, the five domain scores of SDQ and their self-rated academic achievement, using a multiple linear regression model. As covariates, students’ grade, sex and household income were included in the model. Missing data was estimated using the full information maximum-likelihood (FIML). All statistical analyses were processed using Mplus 7.4 [33].

## 3. Results

### 3.1. Consistency of Measurement across Sub-Groups (Measurement Invariance) with JaSC

The result of EFA revealed good model fit with a single-factor solution (RMSEA = 0.023, CFI = 0.996). The first three eigenvalues were 17.3, 1.2 and 0.98, the proportion of the variance of the first components was 54.1%. In the single-factor solution, the geomin rotated factor loadings of 32 items ranged from 0.55 to 0.84 (all loadings were significant at a *p*-value of 0.05; see Table 2). In the two-factor solution, there was no item with a factor loading exceeding 0.3 for the second factor. From these results, we determined that single-factor was the best solution. Table 3 shows the results of further analysis of measurement invariance within the framework of a single-factor solution CFA. The *configural* invariance across sex groups (boys and girls) was demonstrated by adequate model fit statistics (CFI = 0.993, RMSEA = 0.026). The model fit of the *metric* model, in which all factor loadings were constrained equally across the sex groups, was significantly different from the *configural* invariance model (∆χ^2^(∆31) = 51.9, *p* = 0.01) but the differences of CFI and RMSEA were within the criteria of invariance (∆CFI = 0.000, ∆RMSEA = −0.001). The *scalar* model, in which all thresholds were constrained equally across the sex groups, did not fit either, as the fit indices reflected a significantly worse fit than that of the *metric* invariance model (∆χ^2^(∆95) = 103.5, *p* = 0.26, ∆CFI = 0.000, ∆RMSEA = −0.002). Across grade level (elementary and junior high school), the *configural* invariance was also upheld (CFI = 0.994, RMSEA = 0.036). The results of the chi-square difference tests between the *configural* and *metric* models were significant but the differences of CFI and RMSEA were within the criteria of invariance (∆CFI = 0.000, ∆RMSEA = 0.001). The results of chi-square difference tests between the *metric* and *scalar* models were also significant but the differences of CFI and RMSEA were within the criteria of invariance (∆CFI = −0.001, ∆RMSEA = 0.000). These results indicated that the factor structure of JaSC and the factor loadings and thresholds of items were invariant over sex groups (boys and girls) and grade levels (elementary and junior high schools).

### 3.2. Association of the Measured School Climate with Students’ Individual Factors Such as Quality of Life, Bullying Experiences and Behavioral and Emotional Problems

The mean score of 32 items of JaSC as the outcome measure was 3.04 (SD = 0.71) and the median was 3.16. Regarding quality of life of the students, physical, emotional and school functions were significantly related to the JaSC score (β = 0.089, *p* = 0.017; β = 0.069, *p* = 0.041; β = 0.10, *p* < 0.001; β = 0.152, *p* < 0.001, respectively; Table 4). Two out of three forms of involvement in *ijime* (victims and especially bully/victims but not bullies) were negatively related to JaSC scores compared to non-involvement in *ijime* (β = −0.098, *p* = 0.001; β = −0.188, *p* = 0.001 respectively). Of five subscales of SDQ, only the score of the peer relationship problems subscale was negatively associated with the JaSC score (β = −0.107, *p* = 0.025). Of the demographic characteristics, students’ grade was negatively associated with perception of school climate (β = −0.111, *p* = 0.028), which indicated that the older the students, the lower the perception of school climate.

## 4. Discussion

In the present study, we found that the factor structure of the new scale (the JaSC), the factor loadings and the thresholds of each items all pointed toward measurement invariance across grades and gender of the Japanese students. In addition, the regression model indicated that perceived school climate was associated with individual psychological and behavioral properties, especially related to school life, including quality of life in school, bullying victimization and peer problems. These two findings suggest that the JaSC measures school climate among Japanese students consistently and validly and further, provides us with a significant clinical implication that we can aggregate and summarize individual scores into an index indicative of a group values regardless of students’ sex and grade levels.

Many school climate scales developed previously have been found to have a multiple factor structure. Bear et al. [15], however, found that all items of the scale they developed was explained by one general construct of “school climate.” More recently, Leadbeater et al. [34] showed that the sub-dimensions for the existing scale [35] adequately fitted a single factor model. In this study, the JaSC was best explained by a single-factor model with all items loaded on the construct of “school climate.” This result replicated the result of our preliminary study [20]. Bear et al. [15] demonstrated measurement invariance across three grade levels (elementary, middle and high school), racial–ethnic groups and gender. Leadbeater et al. [34] also showed that a single factor model was invariant over time and for child sex. In this study, we also confirmed that the factor structure, factor loadings and thresholds were invariant across sex and grade level.

In the process of examining the external validity, individual psychological and behavioral factors were found to be significantly associated with students’ perception of school climate. These factors contained all subdomains of PedsQL (physical, emotional, social and school functioning). School function especially was most strongly associated with the perception of school climate, which could be evidence of the validity of the JaSC. Zullig et al. [16] also found a significant association between students’ perception of school climate and life satisfaction, self-rated health, and, especially, school satisfaction. A favorable school climate was related to not only quality of life (QOL) in school but also students’ whole QOL. Thus, satisfaction with school life may improve students’ academic achievement and behavioral outcomes.

*Ijime* (bullying) victimization was negatively associated with perceptions of a favorable school climate. It should be noted that involvement in both victim and bully (i.e., “bully/victim”) in the same individual had stronger negative impact on the perception of school climate than involvement only as victim. Although the causality is not clear, the distress that occurs as a result of being involved in *ijime* may induce negative feelings against the school as a whole. Many researchers have reported the association between perceived school climate and bullying [36,37,38]. Flaspohler et al. [39] underscored the importance of both universal bullying prevention programs and school climate improvement strategies as means to reduce a school violence and to promote the well-being of children. It was also reported that the intervention program based on peer tutoring was effective to reduce bullying behavior and at the same time improve the school climate [40].

We investigated the relationship between the scores of SDQ as individual emotional and behavioral traits and the perception of school climate. Among five subscales, only peer problems was related to the perception of school climate. Hung et al. [17] found a negative association between the perception of school climate and emotional and conduct problem measured by SDQ but they did not include other SDQ subscales. In this study, emotional and conduct problems were associated with the perception of school climate in the unadjusted model but the associations were no longer significant in the adjusted model. Higgins-D’Alessandro [41] argued that students with special needs can only benefit from a positive school climate if they feel included and respected by other students. As peer relationships may play a role in the well-being of students with difficulties, a favorable climate of the school as a whole may be particularly important to such students. However, it does not mean that we should focus only on students with special needs. It has been reported that school-wide intervention that does not focus on disturbed children substantially reduces students’ problematic behavior and improved classroom behavior [42].

We obtained information on household income as a factor of family background and included it in the multiple regression model. The result showed that parental income did not significantly affect the students’ perceptions of school climate. This association was also not significant in the crude model (β = 0.01, *p* = 0.79). Consistent with our finding, Hopson and Lee [43] reported that family income did not predict perceptions of climate, although they found that the magnitude of the association between students’ perception of the school climate and their behavior was more pronounced in children from families of lower, than higher, income. It is possible that a positive school climate is more important for students from lower income families. One of the limitations of this study is that the number of participating schools was small and sampling was not random. Further large-scale study using random sampling is required. Second, all the participants belonged to the same city and all were Japanese and thus the findings cannot be generalized outside Japan. In this regard, the participating schools were representative Japanese schools in terms of classroom size, number of students and teachers [44] and the city where the participating schools were located were demographically average for Japan [45]. In fact, the average household income in our sample was quite similar to that in Japan. Thus our findings can be considered representative of Japanese children and comparable to findings in other countries, enabling us to detect points to be addressed in the school climate in Japan. Third, we did not involve school-level factors such as school size and teachers’ characteristics that affect the students’ perception of school climate. Fourth, a large number of items are contained the single-factor scale. Although items involve important suggestions from previous studies, a shortened version of the scale is desirable to conduct surveys at school more easily.

## 5. Conclusions

The present study confirmed the usefulness of the JaSC for measuring students’ perception of school climate in elementary and junior high schools in Japan. We also confirmed that the QOL, especially in school, of the students, involvement in *ijime* and peer problems affect perceptions of the school climate. The identification of these associations is particularly important because it is suggested that the improvement of the school climate can prevent students’ behavioral problems such as *ijime* (bullying). In fact, intervention programs to improve the school climate and prevent bullying have already been developed and their effectiveness confirmed [39,40]. Further study is needed to explore the school-level factors that affect students’ perception of school climate and to test the scale in another country and with different school types to find out the ultimate effectiveness of the scale.

## Figures and Tables

**Table 1:** Demographic characteristics of study samples.

	n (%)	
**Grade level (n = 1399)**		
elementary school	972 (69.5)	
junior high school	427 (30.5)	
**Sex (n = 1396)**		
male	721 (51.5)	
female	675 (48.3)	
**Involvement in *ijime* (n = 1398)**		
not involved	590 (42.2)	
victim	470 (33.6)	
bully	62 (4.4)	
bully/victim	276 (19.7)	
	**mean (SD)**	**range**
School climate scores (n = 1398)	3.10 (0.70)	0.03–4.0
**Scores of self-rated Quality of Life (n = 1397)**		
physical function	91.6 (11.1)	18.8–100
emotional function	80.9 (21.7)	0–100
social function	89.0 (17.2)	0–100
school function	85.3 (17.0)	0–100
**Scores of parent-rated Strengths and Difficulties Questionnaire (n = 1306)**		
emotional symptoms	1.39 (1.73)	0–10
conduct problems	1.71 (1.50)	0–10
hyperactivity–inattention	2.83 (2.06)	0–10
peer relationship problems	1.59 (1.60)	0–10
prosocial behaviors	6.33 (2.11)	0–10
Self-rated academic achievements (n = 1378)	75.1 (17.2)	0–100
Parental income (million yen; n = 1149)	6.05 (2.36)	1–10

**Table 2:** Geomin factor loadings of exploratory factor analysis in each item.

Item Number	Item Contents	Factor Loadings
1	Students feel safe in this school.	0.750
2	Teachers in this school scold the bullies.	0.779
3	Consequences of breaking rules are fair.	0.600
4	School rules are fair.	0.700
5	School rules are applied equally	0.780
6	Students are given clear instructions about how to do their work in classes.	0.650
7	This school makes students enthusiastic about learning	0.670
8	Students in this school often engage in class discussions.	0.660
9	My schoolwork is exciting	0.772
10	Students in this school learn how to change pace or get rid of stress when frustrated.	0.551
11	My teachers make it clear to me when I have misbehaved in class.	0.762
12	Students lean by themselves through experiences in this school how to solve problems such as quarrelling and bullying.	0.681
13	Students in this school learn how to understand other students’ feelings.	0.756
14	Students in this school treat each other with respect.	0.763
15	Students feel happy to accomplish something in cooperation with their classmates.	0.714
16	Students enjoy doing things with each other in school activities.	0.776
17	Teachers let me know when I am doing a good job.	0.715
18	Teachers in this school like students to try unusual projects.	0.708
19	Teachers at my school help us students with our problems.	0.838
20	My teacher makes me feel good about myself.	0.791
21	Students in this school get along well with teachers.	0.775
22	I like this school.	0.773
23	I can participate in a lot of interesting activities at school.	0.722
24	Students in this school put a lot of energy into what they do here.	0.775
25	I feel that I can do well in this school.	0.746
26	Females and males in this school are equally treated with respect.	0.716
27	Students in this school have same opportunity in class to speak and be listened to.	0.773
28	Students get to help decide some of the rules in this school.	0.692
29	Students “different” in any way are treated with respect in this school.	0.795
30	This school is usually clean and tidy.	0.644
31	Parents in this school are involved in discussions about students.	0.619
32	Parents in this school get along well with teachers.	0.703

**Table 3:** Fit statistics for a single-factor solution confirmatory factor analysis testing measurement invariance across sex and grade level.

	∆χ^2^	∆df	*p*-Value	CFI	RMSEA
**Sex (Boys and Girls)**					
*configural*				0.993	0.026
*metric*	51.9	31	0.01	0.993	0.025
*scalar*	103.5	95	0.26	0.993	0.023
**Grade Level (Elementary and Junior High Schools)**					
*configural*				0.994	0.036
*metric*	437.6	31	<0.001	0.994	0.037
*scalar*	436.0	95	<0.001	0.993	0.037

**Table 4:** Linear regression analysis for the mean score of school climate.

	β	95%CI	*p*-Value
**Self-Rated Quality of Life**			
physical function	0.089	0.016, 0.161	0.017
emotional function	0.069	0.003, 0.136	0.041
social function	0.100	0.059, 0.142	<0.001
school function	0.152	0.103, 0.200	<0.001
**Self-Rated Involvement in *ijime* (Non-Involvement is Reference)**			
victims	−0.098	−0.158, −0.039	0.001
bullies	0.007	−0.099, 0.113	0.897
bully/victims	−0.188	−0.300, −0.076	0.001
**Parent-Rated Strengths and Difficulties Questionnaire**			
emotional symptoms	0.004	−0.073, 0.082	0.912
conduct problems	−0.035	−0.081, 0.011	0.136
hyperactivity–inattention	0.012	−0.097, 0.120	0.831
peer relationship problems	−0.107	−0.201, −0.014	0.025
prosocial behaviors	0.009	−0.027, 0.045	0.637
Self-rated academic achievements	0.009	−0.064, 0.082	0.805
Student’s sex ^a^	−0.006	−0.060, 0.049	0.844
Grade ^b^	−0.111	−0.210, −0.012	0.028
Parental income	−0.062	−0.125, 0.001	0.054

β, standardized coefficient; CI, confidence interval; ^a^ “girl” was coded 1 and “boy” was coded 0; ^b^ grades 4 to 6 in elementary school and grades 7 to 9 in junior high school.
